# Effectiveness of Natural Photosensitizers in Antimicrobial Photodynamic Therapy Within Dentistry: A Systematic Review of RCTs

**DOI:** 10.3390/jcm14248894

**Published:** 2025-12-16

**Authors:** Jakub Fiegler-Rudol, Dariusz Skaba, Damian Truchel, Maciej Misiołek, Rafał Wiench

**Affiliations:** 1 Department of Periodontal Diseases and Oral Mucosa Diseases, Faculty of Medical Sciences in Zabrze, Medical University of Silesia, 40-055 Katowice, Poland; dskaba@sum.edu.pl (D.S.); rwiench@sum.edu.pl (R.W.); 2 Specialist Dental Center A.H. Frelich, 44-240 Zory, Poland; 3 Department of Otorhinolaryngology and Laryngological Oncology in Zabrze, Medical University of Silesia, 41-800 Zabrze, Poland

**Keywords:** antimicrobial photodynamic therapy, natural photosensitizers, curcumin, riboflavin, dentistry, randomized controlled trials, oral biofilms, peri-implantitis, periodontitis

## Abstract

**Background:** Antimicrobial photodynamic therapy (aPDT) is a useful adjunct for managing oral biofilm diseases. Natural photosensitizers may be safer and more biocompatible than synthetic ones, but their dental effectiveness is still unclear. **Methods:** A PRISMA compliant review (PROSPERO ID: CRD420251233910) searched PubMed, Embase, Scopus, and the Cochrane Library for randomized controlled trials published from 2015 to 2025 that used natural photosensitizers for aPDT in dental settings. Three reviewers screened studies, extracted data, and assessed bias with a nine-domain tool adapted for photodynamic therapy. **Results:** Eleven of 249 records met the established criteria. Natural photosensitizers included curcumin, riboflavin, phycocyanin, chlorophyll derivatives, and plant extracts, tested in periodontitis, peri-implant mucositis, denture stomatitis, caries-related biofilms, and general oral decontamination. Most trials showed short-term microbial reductions and modest clinical gains, with performance comparable to chlorhexidine, methylene blue, or standard care. Adverse effects were minimal. Study quality was generally good, but wide variation in photosensitizer type, light settings, and outcomes, and short follow-up periods hindered meta-analysis and limited conclusions about long-term effectiveness. **Conclusions:** Natural photosensitizer-based aPDT appears effective and safe as an adjunct, offering consistent short-term microbiological improvements. Current evidence does not support replacing established antimicrobial approaches. Larger, well-controlled trials with standardized methods and longer follow-up periods are needed to define best practice and clarify the role of aPDT in routine dentistry.

## 1. Introduction

### 1.1. Background

The rise of antimicrobial resistance has created major challenges across healthcare, including dentistry, where conventional antimicrobial agents are becoming less effective against biofilm-related oral pathogens [1]. Conditions such as periodontal disease, peri-implantitis, peri-implant mucositis, and other infections driven by complex biofilms often respond unpredictably to mechanical debridement and standard antiseptics, which has increased interest in adjunctive therapies capable of enhancing microbial control [2]. Antimicrobial photodynamic therapy (aPDT) has emerged as one such option. It relies on the activation of a photosensitizer by light in the presence of oxygen to generate reactive oxygen species that selectively damage microbial cells while leaving host tissues largely unaffected [3]. This targeted mode of action makes aPDT particularly suitable for managing biofilms that are otherwise difficult to eradicate. Clinical research has historically focused on synthetic photosensitizers, which offer consistent photochemical behavior and potent antimicrobial effects [4]. Increasing attention, however, has turned toward natural photosensitizers derived from plant and microbial sources, driven by concerns regarding cytotoxicity, environmental impact, cost, and the risk of microbial tolerance associated with some synthetic compounds [4,5,6,7,8,9,10]. Natural agents such as curcumin, chlorophyll derivatives, and riboflavin (vitamin B_2_) possess advantageous properties, including biocompatibility, biodegradability, low toxicity, and inherent antimicrobial, anti-inflammatory, or antioxidant activity [4,6]. Their broader adoption aligns with growing interest in sustainable and patient-friendly therapeutic approaches [4,5,6,7,8,9,10,11,12]. Despite these theoretical and laboratory supported advantages, the clinical integration of natural photosensitizers remains limited. Many natural compounds have variable photochemical characteristics and issues related to solubility, stability, and bioavailability that can compromise clinical performance [6,7]. Advances in pharmaceutical formulations and nanocarrier-based delivery systems have begun to address these limitations, yet these innovations require confirmation through well-designed clinical trials [7]. There is also no consensus on optimal treatment parameters, including photosensitizer concentration, light wavelength, irradiation time, or dosing frequency, which contributes to inconsistent outcomes across studies [5]. This topic has clinical importance because dentistry is increasingly constrained by antimicrobial resistance and by patient demand for safer and more natural adjunctive therapies. Although laboratory findings have been promising, no comprehensive synthesis of contemporary randomized controlled trials has focused specifically on the clinical performance of natural photosensitizers in aPDT. A targeted evaluation of this evidence is needed to clarify its utility, identify knowledge gaps, and support evidence-based decision making.

### 1.2. Objectives

Given these considerations, a comprehensive systematic review focusing exclusively on randomized controlled trials is warranted to critically evaluate the current evidence regarding the efficacy of natural photosensitizers in antimicrobial photodynamic therapy for dental applications [1,5]. This review seeks to clarify clinical and microbiological outcomes, identify strengths and limitations in the existing literature, and provide informed recommendations to guide future research and facilitate integration of natural photosensitizers into evidence-based dental practice.

## 2. Methods

This systematic review followed established methodological standards for evidence synthesis and was conducted in accordance with PRISMA 2020 [13]. The protocol was prospectively registered in the PROSPERO database (registration number CRD420251233910) [14].

### 2.1. Focused Question

In dental patients receiving antimicrobial photodynamic therapy (Population), does the use of natural photosensitizers (Intervention), compared with synthetic photosensitizers or no photodynamic therapy (Comparison), improve clinical or microbiological outcomes related to oral infections (Outcome) [15]? Primary outcomes were quantitative measures of microbial reduction, including bacterial or fungal load and biofilm viability. Secondary outcomes included probing depth, bleeding on probing, clinical attachment level, oral mucositis severity, resolution of denture-related stomatitis, and any reported adverse effects.

### 2.2. Search Strategy

A comprehensive electronic search was performed across PubMed, Embase, Scopus, and the Cochrane Library to identify studies evaluating aPDT mediated by natural photosensitizers within dentistry. Three independent reviewers executed searches using predefined combinations of MeSH terms and free-text keywords associated with antimicrobial photodynamic therapy, natural photosensitizers, and dental applications. Searches were limited to English language studies published from 2015 to 2025, with no geographical restrictions. The screening process was performed between July and September 2025. Screening was performed in two phases: first, title and abstract screening to identify potentially eligible records, and second, full-text review based on predefined inclusion and exclusion criteria. Reference lists of all included studies were manually screened to identify additional eligible articles. The search aimed to capture clinical, in vitro, and ex vivo research examining the effectiveness of natural photosensitizers used in antimicrobial photodynamic therapy within dental disciplines. The search syntax is shown in Table 1.

### 2.3. Study Selection Process

Study selection was conducted independently by three reviewers. Titles and abstracts were screened according to predefined inclusion and exclusion criteria. Eligible studies included clinical trials, in vitro studies, ex vivo studies, and comparative research examining antimicrobial photodynamic therapy using natural photosensitizers in dental contexts. Included studies needed to report measurable microbiological or clinical outcomes, such as bacterial load reduction, periodontal pocket improvement, caries-related microbial changes, biofilm disruption, or therapeutic outcomes in endodontics, periodontics, or oral surgery. Exclusion criteria included narrative reviews, editorials, conference abstracts, in vivo animal studies without dental relevance, studies using only synthetic photosensitizers, and publications not available in English. Duplicates were removed, and discrepancies were resolved through discussion.

### 2.4. Data Extraction

Three reviewers independently extracted data using a standardized form. Extracted variables included author, year of publication, study design, type of natural photosensitizer used, light source parameters, targeted microorganisms or clinical condition, sample size, outcome measures, and main findings. Microbiological and clinical outcomes were recorded where applicable, including bacterial reduction rates, changes in clinical inflammatory indices, biofilm disruption levels, healing parameters, and follow-up durations. Details regarding photosensitizer preparation, concentration, and activation wavelength were also extracted.

### 2.5. Risk of Bias and Quality Assessment

Methodological quality was independently evaluated by three reviewers using an adapted appraisal tool for photodynamic therapy research. The following domains were assessed:

1. Clear description of the natural photosensitizer, including preparation and concentration.
2. Specification of light source parameters such as wavelength, power density, and irradiation time.
3. Measurement of clinically or microbiologically relevant outcomes.
4. Inclusion of appropriate comparator groups, such as untreated controls or synthetic photosensitizers.
5. Clear inclusion and exclusion criteria for sample selection.
6. Consideration of bias control measures including randomization, calibration, or blinding, where applicable.
7. Transparency and reproducibility of statistical analysis.
8. Completeness of outcome reporting, including adverse effects or limitations.
9. Disclosure of funding and potential conflicts of interest.

Each domain received a score of 1 or 0, giving total possible scores of 0 to 9. Studies scoring 7 to 9 were classified as low risk, 4 to 6 as moderate risk, and 0 to 3 as high risk. Disagreements were resolved through discussion or by a fourth reviewer. The assessment followed methodological guidance from the Cochrane Handbook for Systematic Reviews of Interventions [16].

### 2.6. Assessment of the Quality of Evidence

A structured review of the evidence for each outcome was carried out using the Grading of Recommendations Assessment, Development, and Evaluation (GRADE) framework, which guides the appraisal of research certainty [17]. Evidence was sorted into four levels: high, moderate, low, or very low quality. Because judging GRADE elements can involve interpretation, three authors independently reviewed the criteria. Any differing views were resolved through discussion, supported by calculating agreement with Cohen’s k test.

## 3. Results

### 3.1. Study Selection

**Figure 1:**
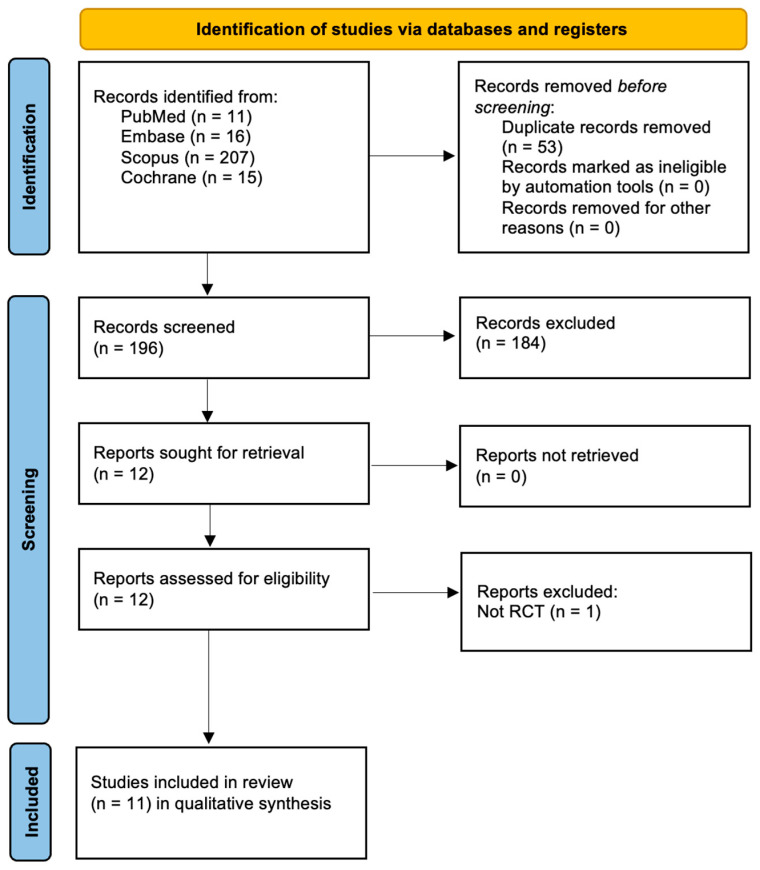
PRISMA 2020 Flowchart.

The database search yielded 249 records, including 11 from PubMed, 16 from Embase, 207 from Scopus, and 15 from the Cochrane Library. After removing 53 duplicates, 196 records remained for screening. Title and abstract screening resulted in the exclusion of 184 records, leaving 12 reports for retrieval. All 12 reports were successfully retrieved and assessed for eligibility. One report was excluded for not being a randomized controlled trial, resulting in 11 studies being included in the qualitative synthesis. This is shown in Figure 1.

### 3.2. Assessment of the Risk of Bias

The assessment shows that nearly all included studies demonstrated strong methodological quality, with most scoring eight or nine out of nine items and falling into the low-risk category. Only one study was rated as medium risk. Despite this outlier, the overall evidence base is characterized by consistent low risk ratings, suggesting that the findings drawn from these studies provide a generally reliable and robust set of data. This is shown in Table 2.

### 3.3. Assessment of the Quality of Evidence

This summary provides a clearer indication of how confidently the current evidence can be interpreted and is detailed in Table 3.

### 3.4. Characteristics of the Included Studies

Across the included studies, sample designs varied widely, with research conducted in Iran, Saudi Arabia, and multiple regions of Brazil. Several studies used grouped laboratory or clinical samples such as implants, molars, tooth blocks, or adult participants, while others followed split mouth- or device-based designs. Sample sizes ranged from small controlled groups to larger clinical sets, and in some cases initial enrollment differed from final completion numbers. Overall, the collection of studies reflects broad geographic coverage and heterogeneous methodologies suited to their respective research aims [18,19,20,21,22,23,24,25,26,27,28] (Table 4).

### 3.5. Main Outcomes from Studies

When interpreting the results, outcomes from low-risk studies were prioritized. Findings from trials with moderate risk or incomplete reporting were considered supportive but not definitive. This approach allowed balanced interpretation while acknowledging methodological variability. Across the studies, most interventions using curcumin, riboflavin, toluidine blue, or Photogem combined with light sources produced meaningful antimicrobial or clinical benefits, often outperforming controls. Several investigations showed strong reductions in bacterial or fungal counts with photodynamic approaches, particularly when specific photosensitizers or light parameters were paired effectively [18,21,24,25,26,27]. Some trials demonstrated clinical improvements such as reduced mucositis, pain, periodontal parameters, or *Candida* levels, with aPDT frequently matching or exceeding conventional treatments like chlorhexidine or nystatin [19,20,23,28]. A number of studies reported specific limitations associated with natural photosensitizers, including reduced bond strength under certain adhesive protocols [22], variable antimicrobial effectiveness depending on photosensitizer–light pairing [18,21,27], and inconsistent persistence of clinical improvements [24,28]. Despite these shortcomings, the overall evidence supports the antimicrobial and therapeutic potential of light-based therapies when appropriate parameters are selected. Patient acceptance and user experience were not consistently assessed across the included trials. The limited available information indicates minimal discomfort and few adverse effects, yet key aspects such as taste, staining potential, ease of application, and overall satisfaction have not been systematically evaluated. Future studies should incorporate structured patient-reported measures to better characterize usability and acceptability.

Across the included trials, natural photosensitizers demonstrated meaningful antimicrobial or clinical effects across diverse dental applications. Afrasiabi et al. [18] showed that all tested photosensitizer–light combinations significantly reduced *A. actinomycetemcomitans* on implant surfaces, with LED-mediated aPDT producing the lowest CFU counts. In restorative dentistry, AlSunbul et al. [19] found that methylene blue-mediated aPDT achieved the highest bonding values and strongest antibacterial activity, while curcumin-mediated aPDT produced the greatest long-term 4-point bending strength. In the management of oral mucositis, de Cássia Dias Viana Andrade et al. [20] reported that both photobiomodulation- and curcumin-based aPDT reduced *Candida* levels and improved symptoms, with aPDT showing earlier clinical benefit. Donato et al. [21] demonstrated immediate microbial reductions with curcumin and Photogem, though only curcumin sustained the effect at 24 h. Hashemikamangar et al. [22] showed that aPDT did not impair bonding in self-etch protocols, with phycocyanin producing the highest bond strength. In periodontitis, Ivanaga et al. [23] found that all groups improved over time, though clinical attachment gain occurred only in the LED and aPDT groups at three months. Labban et al. [24] showed that curcumin- and rose bengal-mediated aPDT achieved antifungal effects comparable to nystatin in smokers with denture stomatitis. Leite et al. [25] observed significant short-term reductions in salivary CFU following curcumin-based aPDT compared with light alone or curcumin alone. Panhóca et al. [26] demonstrated enhanced antimicrobial activity when surfactant was added to curcumin-mediated aPDT, performing similarly to chlorhexidine. A related in situ study by Panhóca et al. [27] confirmed that curcumin-mediated aPDT reduced *S. mutans* biofilm, though Photogem produced the largest reduction. Paschoal et al. [28] reported that curcumin-mediated c PACT reduced gingival bleeding at one month but did not improve plaque levels compared with chlorhexidine varnish or placebo (Table 5 and Table 6).

## 4. Discussion

### 4.1. Results in the Context of Other Evidence

Antimicrobial photodynamic therapy (aPDT) in dentistry utilizes photosensitizers activated by light to generate reactive oxygen species, leading to microbial cell death. Natural photosensitizers such as curcumin, riboflavin, phycocyanin, chlorophyll derivatives, and certain plant extracts have demonstrated significant antimicrobial efficacy against oral pathogens, including those in biofilms associated with periodontitis, peri-implantitis, and denture-related infections [29,30,31,32,33]. In vitro and clinical studies show that natural photosensitizers, when combined with appropriate light sources such as LED or blue light, can reduce bacterial and fungal colony counts and disrupt biofilm structure. Curcumin and riboflavin have shown antimicrobial effects comparable to conventional disinfectants in peri-implantitis and denture biofilm models, though complete biofilm eradication is rarely achieved [32,33,34]. Advantages of natural photosensitizers include low toxicity, biocompatibility, and environmental sustainability. However, limitations such as poor solubility and bioavailability persist, prompting ongoing research into nanocarrier-based delivery systems to enhance clinical effectiveness [31,35]. Despite promising results, heterogeneity in protocols and insufficient long-term clinical data prevent standardized recommendations. Additional high-quality randomized controlled trials are needed to establish optimal dosing, irradiation parameters, and long-term outcomes [32,36]. Natural photosensitizers are effective adjuncts in aPDT for dental infections, offering antimicrobial and anti-biofilm activity with favorable safety profiles, but require further translational research before routine clinical adoption [1,6,36,37,38,39]. Current evidence from randomized controlled trials comparing natural photosensitizers with conventional agents in aPDT indicates that natural agents such as curcumin, riboflavin, and 5 aminolevulinic acid are effective adjuncts for reducing microbial load and improving clinical indices in periodontal and peri-implant diseases, but their long-term efficacy and superiority over conventional agents remain unproven due to methodological heterogeneity and limited follow-up data [7,30,31]. In peri-implantitis, systematic reviews of randomized trials show that natural photosensitizers achieve reductions in bacterial viability, probing depth, and bleeding on probing comparable to conventional agents such as toluidine blue, though complete biofilm eradication is rarely achieved. Curcumin and riboflavin demonstrate similar short-term clinical improvements, but toluidine blue remains the most effective for sustained outcomes based on meta-analyses [30,31]. Most studies report favorable safety profiles and minimal adverse effects for natural agents [30]. For periodontitis, adjunctive aPDT, regardless of photosensitizer type, provides modest improvements in probing depth and clinical attachment level at six months. These changes are not consistently clinically significant, and the certainty of the evidence is very low due to risk of bias and small sample sizes [37]. Long-term data beyond six months are sparse, and standardized protocols for natural photosensitizers are lacking [30,31,32,33,34,35,36,37]. Natural photosensitizers are effective adjuncts in aPDT for short-term microbial and clinical improvements, but they do not outperform conventional agents in long-term efficacy. Further large-scale, well designed randomized controlled trials with standardized protocols and extended follow-up periods are needed to establish aPDT’s role in routine dental practice [30,31,37].

### 4.2. Limitations of the Evidence

Although the included trials indicate that natural photosensitizers can reduce microbial load and yield short-term clinical improvements, several limitations weaken the overall certainty of the evidence. Protocols differed markedly across studies, including variations in photosensitizer type, concentration, solvent, light wavelength, power, and irradiation time. Reporting of dosimetry was often incomplete, which limited reproducibility and prevented direct comparison or pooled analysis. Most trials involved small samples, often below thirty participants or test units per group, reducing statistical power and increasing the likelihood of type two errors. Follow-up periods were generally short, with few studies extending beyond three months, so the durability of clinical or microbiological benefits remain unclear. Comparator groups also varied widely, ranging from synthetic photosensitizers to chlorhexidine or no adjunctive treatment, complicating assessment of relative effectiveness. Safety reporting was inconsistent. Many studies did not document adverse effects, staining, or patient acceptability, leaving the full risk profile of natural photosensitizers uncertain. Methodological details were incompletely described in several trials, including randomization and examiner calibration, adding further uncertainty. Finally, many microbial assessments relied on simplified models rather than complex multispecies biofilms, limiting insight into the ecological impact of treatment. These constraints collectively restrict the strength, generalizability, and long-term implications of the current evidence base.

### 4.3. Limitations of the Review Process

Although this review followed PRISMA guidelines and a comprehensive search strategy across four major databases was implemented, the review process itself presents limitations that should be acknowledged. Restricting searches to English language publications may have led to the exclusion of eligible non-English trials. The decision to limit the time frame from 2015 to 2025 ensured contemporary relevance but may have excluded earlier foundational RCTs on natural photosensitizers. Study selection and data extraction were performed independently by three reviewers, yet the interpretation of incomplete reporting within some primary studies could have introduced subjective judgment. While efforts were made to minimize selection bias, grey literature, unpublished trials, and non-indexed studies may have been missed. A pooled quantitative analysis was not possible due to substantial variation in study design, intervention protocols, and outcome metrics. These factors prevented generation of a common effect size and restricted synthesis to a qualitative approach. Another limitation arises from incomplete information within several primary trials. Missing data on irradiation parameters, photosensitizer preparation, and baseline microbial characteristics limited the precision of extraction and reduced the ability to fully assess methodological rigor. Despite the use of a structured quality assessment tool, variability in study design made uniform scoring challenging. Finally, although the review aimed to distinguish between clinical, in situ, and laboratory conditions, the overlap between these categories in some experimental designs may have affected the consistency of classification.

### 4.4. Implications for Practice, Policy, and Future Research

The current body of evidence suggests that natural photosensitizers have promising antimicrobial activity and may serve as useful adjuncts in periodontal therapy, peri-implant care, endodontic disinfection, and management of denture-associated infections. Curcumin, riboflavin, and chlorophyll derivatives are among the most studied agents and have demonstrated reductions in microbial viability and modest improvements in clinical indices. While these findings support their potential incorporation into clinical workflows, the absence of standardized dosing protocols and the limited availability of commercial formulations adapted for dental use restrict immediate widespread adoption. For clinical practice, natural photosensitizers may be considered in selected cases as adjunctive agents, particularly when patients prefer natural compounds or when conventional antiseptics are contraindicated. However, practitioners should be cautious due to variability in treatment parameters and the limited evidence for long-term outcomes. Current data do not support replacing synthetic photosensitizers or established antimicrobials with natural alternatives.

From a policy standpoint, regulatory bodies may need to develop guidelines that encourage consistent reporting of photophysical parameters and manufacturing standards for natural photosensitizers. More robust regulation would support safer translation of these agents into clinical products and foster industry research into optimized formulations. Future research should prioritize large-scale randomized controlled trials with standardized photosensitizer concentrations, irradiation protocols, and clinically relevant outcomes. Trials should include follow-up periods beyond six months to determine the durability of microbiological and clinical benefits. Research into novel formulations, especially nanocarrier-assisted systems, may help improve solubility and tissue penetration. Comparative trials between natural and synthetic photosensitizers, as well as cost-effectiveness analyses, will also be essential to define their role in routine practice. Improved reporting of adverse effects, staining potential, patient acceptability, and operator handling characteristics will further enhance clinical relevance.

The choice of natural photosensitizer can be adjusted to the characteristics of the oral infection and the nature of the biofilm. Curcumin has broad antibacterial and anti-inflammatory activity and is effective against superficial biofilms due to its strong absorption in the blue spectrum and rapid photoreactivity, making it suitable for periodontal and caries-related applications [4,30,32]. Riboflavin functions well in moist oral environments and demonstrates reliable activation with blue light, which supports its use in peri-implant mucositis and other situations requiring consistent penetration through biofilm matrices [30,31]. Chlorophyll derivatives and phycocyanin absorb strongly in the red range, providing deeper tissue penetration and potential advantages for thicker or more mature biofilms often associated with peri-implantitis and denture-related infections [4,33]. These differences suggest that photosensitizer choice may be optimized by considering microbial composition, lesion depth, and the optical properties of the target site. Future trials should directly compare natural agents across specific clinical scenarios to determine infection-specific selection strategies.

## 5. Conclusions

This review shows that natural photosensitizers such as curcumin, riboflavin, phycocyanin, and chlorophyll derivatives can provide short-term microbial reduction and modest clinical improvements across various dental conditions. Their safety profile appears favorable, but evidence remains limited by heterogeneous protocols, small sample sizes, incomplete reporting of dosimetry, and short follow-up periods. Natural photosensitizers can currently be considered useful adjuncts but not substitutes for established antimicrobial strategies. Further well-designed randomized trials with standardized interventions and longer follow-up periods are needed to clarify their long-term effectiveness and define their role in routine dental care.

## Figures and Tables

**Table 1:** Search syntax used in the study.

Source	Search Syntax	Filters	N
PubMed	(photodynamic therapy[MeSH] OR photodynamic therapy[Title/Abstract] OR aPDT[Title/Abstract]) AND (natural photosensitizer*[Title/Abstract] OR plant derived photosensitizer*[Title/Abstract] OR curcumin[Title/Abstract] OR hypericin[Title/Abstract] OR quercetin[Title/Abstract] OR “gallic acid”[Title/Abstract] OR “aloe emodin”[Title/Abstract] OR chlorophyll[Title/Abstract] OR chlorophyllin[Title/Abstract] OR psoralen[Title/Abstract] OR psoralens[Title/Abstract] OR furanocoumarin*[Title/Abstract] OR polyacetylene*[Title/Abstract] OR thiophene*[Title/Abstract] OR anthraquinone*[Title/Abstract]) AND (dentistry[MeSH] OR dental[Title/Abstract] OR oral[Title/Abstract] OR odontolog*[Title/Abstract])	RCT, 2015 to 2025	11
Embase	(‘photodynamic therapy’/exp OR ‘photodynamic therapy’:ti,ab OR aPDT:ti,ab) AND (“natural photosensitizer*”:ti,ab OR “plant derived photosensitizer*”:ti,ab OR curcumin:ti,ab OR hypericin:ti,ab OR quercetin:ti,ab OR “gallic acid”:ti,ab OR “aloe emodin”:ti,ab OR chlorophyll:ti,ab OR chlorophyllin:ti,ab OR psoralen*:ti,ab OR furanocoumarin*:ti,ab OR polyacetylene*:ti,ab OR thiophene*:ti,ab OR anthraquinone*:ti,ab) AND (‘dentistry’/exp OR dental:ti,ab OR oral:ti,ab OR odontolog*:ti,ab)	RCT, 2015 to 2025	16
Scopus	TITLE-ABS-KEY (“photodynamic therapy” OR aPDT) AND TITLE-ABS-KEY (“natural photosensitizer*” OR “plant derived photosensitizer*” OR curcumin OR hypericin OR quercetin OR “gallic acid” OR “aloe emodin” OR chlorophyll OR chlorophyllin OR psoralen* OR furanocoumarin* OR polyacetylene* OR thiophene* OR anthraquinone*) AND TITLE-ABS-KEY (dental OR oral OR dentistry OR odontolog*)	Article, 2015 to 2025	207*
Cochrane Library	([mh “Photochemotherapy”] OR “photodynamic therapy”:ti,ab OR aPDT:ti,ab) AND (“natural photosensitizer*”:ti,ab OR “plant derived photosensitizer*”:ti,ab OR curcumin:ti,ab OR hypericin:ti,ab OR quercetin:ti,ab OR “gallic acid”:ti,ab OR “aloe emodin”:ti,ab OR chlorophyll:ti,ab OR chlorophyllin:ti,ab OR psoralen*:ti,ab OR furanocoumarin*:ti,ab OR polyacetylene*:ti,ab OR thiophene*:ti,ab OR anthraquinone*:ti,ab) AND ([mh Dentistry] OR dental:ti,ab OR oral:ti,ab OR odontolog*:ti,ab)	No additional filters	15

* Scopus does not have an ‘RCT’ filter.

**Table 2:** The results of the quality assessment and risk of bias assessment across the studies.

Study	1	2	3	4	5	6	7	8	9	Total	Risk
Afrasiabi et al. 2022 [18]	1	1	1	1	1	0	1	1	1	8	Low
AlSunbul et al. 2023 [19]	1	1	1	1	1	1	1	1	1	9	Low
de Cássia Dias Viana Andrade et al. 2022 [20]	1	1	1	1	1	1	1	1	1	9	Low
Donato et al. 2017 [21]	1	1	1	1	1	0	1	1	0	7	Low
Hashemikamangar et al. 2022 [22]	1	1	1	1	1	1	1	1	1	9	Low
Ivanaga et al. 2019 [23]	1	1	1	1	1	1	1	1	1	9	Low
Labban et al. 2021 [24]	0	0	0	0	1	1	0	1	1	1	Medium
Leite et al. 2014 [25]	1	1	1	1	1	1	1	1	1	9	Low
Panhóca et al. 2016a [26]	1	1	1	1	1	1	1	1	1	9	Low
Panhóca et al. 2016b [27]	1	1	1	1	1	1	1	1	1	9	Low
Paschoal et al. 2015 [28]	1	1	1	1	1	1	1	1	1	9	Low

**Table 3:** Summary of findings (SoFs) and quality of evidence (GRADE).

Outcome	Number of Studies	Number of Patients/Samples	Study Design	Risk of Bias	Inconsistency	Indirectness	Imprecision	Publication Bias	Quality of Evidence	Importance
Microbial reduction (CFU/mL)	5	~250 samples/patients	RCTs and in vitro experiments	Moderate (mix of in vitro and clinical)	Moderate (effect size varies widely)	Moderate (lab findings ≠ clinical environment)	Low	None detected	Moderate	Critical
Bond strength (SBS/µTBS/4P-BS)	2	220 samples	In vitro RCTs	Low	Low	Moderate (lab outcomes; not clinical longevity)	Moderate (sample variability)	None detected	Moderate	Important
Probing depth (PD)	1	25 patients	RCT	Low	Low	Low	Moderate (small N)	None detected	Moderate	Critical
Clinical attachment level (CAL)	1	25 patients	RCT	Low	Low	Low	Moderate	None detected	Moderate	Critical
Gingival inflammation (BOP/GI/PI)	2	70 patients	RCTs	Low	Low	Low	Moderate	None detected	Moderate	Important

**Table 4:** A general overview of the studies.

Study	Geographical Location	Total Sample Size
Afrasiabi et al. 2022 [18]	Iran	60 implants
AlSunbul et al. 2023 [19]	Saudi Arabia	100 molars
de Cássia Dias Viana Andrade et al. 2022 [20]	Brazil	30 patients
Donato et al. 2017 [21]	Brazil	50 volunteers
Hashemikamangar et al. 2022 [22]	Iran	120 tooth blocks
Ivanaga et al. 2019 [23]	Brazil	25 patients
Labban et al. 2021 [24]	Saudi Arabia	45 participants
Leite et al. 2014 [25]	Brazil	27 adults
Panhóca et al. 2016a [26]	Brazil	24 patients
Panhóca et al. 2016b [27]	Brazil	18 participants
Paschoal et al. 2015 [28]	Brazil	45 initial; 30 completed (final sample)

**Table 5:** Main outcomes and details from each study.

Study	Study Groups	Main Outcomes
Afrasiabi et al. 2022 [18]	CUR 10 mg/mL + blue diode laser (450 nm, 60 s) CUR 10 mg/mL + blue LED (430–460 nm, 60 s) RB 10 mg/mL + blue diode laser (450 nm, 60 s) RB 10 mg/mL + blue LED (430–460 nm, 60 s) TBO 0.1 mg/mL + red diode laser (635 nm, 60 s) TBO O 0.1 mg/mL + red LED (630 ± 10 nm, 20 s) PC 2 mg/mL + red diode laser (635 nm, 60 s) PC 2 mg/mL + red LED (630 ± 10 nm, 20 s) No treatment−control 0.2% CHX for 60 s	All disinfection methods produced significant reductions in *A. actinomycetemcomitans* on dental implant surfaces compared with the control. The LED-based aPDT group showed the lowest CFU/mL, outperforming diode laser treatment. LED-mediated aPDT appears to be a more effective adjunct for implant surface disinfection.
AlSunbul et al. 2023 [19]	IPC only 2% CHX gel 6% NaOCl MB-mediated aPDT CUR-mediated aPDT	At baseline and after 12 months, MB-mediated aPDT produced the highest SBS and μTBS values, while CUR-mediated aPDT yielded the highest 4P-BS after long-term storage. MB-mediated aPDT showed the strongest antibacterial activity against *S. mutans*. aPDT, particularly MB-mediated, outperformed 2 percent chlorhexidine gel and 6% NaClO for cavity disinfection in IPC-treated permanent molars.
de Cássia Dias Viana Andrade et al. 2022 [20]	Control group (nystatin) PBM group, aPDT group (450-nm blue LED + CUR).	Both active treatments reduced *Candida* levels at 21 and 30 days, but not at 7 or 14 days. Mucositis worsened in the control group after day 14, while it improved in the aPDT group beginning at day 21. Both PBM and aPDT reduced mucositis and pain, with aPDT showing earlier clinical improvement and greater antifungal effectiveness.
Donato et al. 2017 [21]	For each PS (CUR or Photogem): Water control, PS-only control, light-only control, PS 25 mg/L + light, PS 100 mg/L + light.	All treatments produced an immediate microbial reduction after PDI, regardless of the photosensitizer used. After 24 h, only natural CUR maintained the reduction, while Photogem showed a return to baseline CFU levels. Natural CUR demonstrated better sustained efficacy and appears to be a more viable photosensitizer, supporting PDI as a promising method for oral microbial reduction.
Hashemikamangar et al. 2022 [22]	Self-etch vs total-etch × (control, TBO-aPDT, PC-aPDT) × (with/without thermocycling).	aPDT did not significantly affect bonding strength in self-etch groups, but total-etch groups showed a significant reduction after aPDT. The phycocyanin self-etch group achieved the highest bond strength, and thermocycling did not significantly affect dentin bond strength except in the control total-etch group. aPDT with TBO or phycocyanin did not harm bonding to sound dentin using a universal adhesive in self-etch mode, with phycocyanin recommended as the preferred photosensitizer.
Ivanaga et al. 2019 [23]	SRP only. CUR group: SRP + irrigation + CUR solution (100 mg/L). LED group: SRP plus LED irradiation aPDT group: SRP plus CUR irrigation (100 mg/L), 1-min pre-irradiation, and LED irradiation (InGaN, 465–485 nm, 60 s).	Intergroup comparisons showed no differences among groups in PD, GR, CAL, BOP, or PI at baseline, 3 months, or 6 months. All groups showed significant reductions in PD and BOP over time, while CAL gain occurred only in the aPDT and LED groups at three months. SRP combined with CUR-mediated aPDT or LED irradiation provided short-term CAL improvement in treating residual pockets in patients with type 2 diabetes.
Labban et al. 2021 [24]	RB-mediated PDT CUR-mediated PDT Nystatin	Groups I and II showed significant reductions in CFU counts after treatment and at 12 weeks, with clinical efficacy rates of 53 percent, 51 percent, and 49 percent for Groups I, II, and III. CUR- and RB-mediated PDT performed comparably to topical nystatin in treating denture stomatitis in smokers.
Leite et al. 2014 [25]	PDT group: CUR + blue light Light group: blue light only, no CUR CUR group: CUR only, no light	The PDT group showed significant CFU reductions immediately after treatment and at 1 h and 2 h compared with pretreatment, while the light group showed no change. The CUR only group showed a temporary increase in CFU at 1 h, returning to baseline by 2 h. PDT produced significantly greater microbial reduction than both light and CUR alone through 2 h, indicating that the blue LED–CUR protocol can reduce salivary microorganisms, though further protocol refinement is needed.
Panhóca et al. 2016a [26]	Blue light only. CUR + blue light. CUR with surfactant + blue light. CHX treatment.	Significant log reductions were observed in the PDT, PDT + S, and chlorhexidine groups, with the greatest decreases in the PDT + S and chlorhexidine treatments. Survival rates were significantly lower in the PDT + S and chlorhexidine groups than in all other conditions, with no difference between these two groups. The findings suggest that adding surfactant enhances aPDT effectiveness, making it a useful adjunct for oral decontamination.
Panhóca et al. 2016b [27]	No photosensitizer and no light (PS–L–). CUR without light (PS + L–). CUR with light (PS + L+). Photogem^®^ with light (PS + L+).	aPDT produced significant reductions in CFU counts, with Photogem plus light showing the greatest decrease. CUR alone, CUR plus light, and Photogem plus light all reduced *S. mutans* biofilm compared with the control, though differences among groups were small. Percentage reductions were 8% for CUR, 15 percent for CUR plus light, and 18% for Photogem plus light.
Paschoal et al. 2015 [28]	2% CHX varnish group Placebo varnish group c-PACT PDT (CUR + LED)	PI did not differ among groups at baseline or 1 month. At 3 months, Group III showed higher PI (1.52 ± 0.51) than Group I (0.91 ± 0.75) and Group II (1.03 ± 0.51), and all groups exhibited increased plaque compared with earlier periods (*p* ≤ 0.05). GBI decreased significantly at 1 month in Groups I and III. Photodynamic treatment with CUR did not reduce plaque accumulation.

CUR—curcumin; DL—diode laser; LED—light-emitting diode; RB—riboflavin; TBO—toluidine blue O; PC—phycocyanin; CHX—chlorhexidine; PS—photosensitizer; CFU—colony-forming units; IPC—interim polycarbonate crown; NaOCl—sodium hypochlorite; MB—methylene blue; aPDT—antimicrobial photodynamic therapy; SBS—shear bond strength; μTBS—microtensile bond strength; 4P-BS—four-point bending strength; PBM—photobiomodulation; PDT—photodynamic therapy; SRP—scaling and root planing; CAL—clinical attachment loss; BOP—bleeding on probing; RB—rose bengal; SDS—sodium dodecyl sulfate; RB—rose bengal; c PACT—photodynamic antimicrobial chemotherapy using curcumin.

**Table 6:** Physical parameters of light sources.

Study	Light Type	Wavelength (nm)	Intensity (mW/cm^2^)	Irradiation Time (s)	Irradiated Area (cm^2^)	Fluence (J/cm^2^)
Afrasiabi et al. 2022 [18]	Blue DL	450	500	60	0.5	60
	Blue LED	430–460	1000 ± 100	60	0.5	60
	Red DL	635	500	60	0.5	60
	Red LED	630 ± 10	2000–4000	20	0.5	60
AlSunbul et al. 2023 [19]	LED curing light	385–515	1200	-	-	-
de Cássia Dias Viana Andrade et al. 2022 [20]	Blue LED	450	67	600	-	20.1
	Red laser	660	100	3	0.25	1.2
Donato et al. 2017 [21]	Blue LED	450	100,000	360	-	-
	Red LED	630	100,000	360	-	-
Hashemikamangar et al. 2022 [22]	Diode laser	635	340	180	-	-
Ivanaga et al. 2019 [23]	LED (InGaN)	465–485	100	60	0.78	6
Labban et al. 2021 [24]	Blue LED	455	102	-	-	-
Leite et al. 2014 [25]	Blue LED	455 ± 30	600	300	0.6	200
Panhóca et al. 2016a [26]	Extra-oral Blue LED	450 ± 10	80	-	-	14
	Intra-oral Blue LED	450 ± 10	472	-	-	85
Panhóca et al. 2016b [27]	Blue LED	450 ± 5	764	60	0.25	45.84
	Red LED	630 ± 5	381	120	0.25	45.72
Paschoal et al. 2015 [28]	Blue LED	450	165	192	0.28	96

DL—diode laser; LED—light-emitting diode; InGaN—indium gallium nitride.

## Data Availability

No new data were created or analyzed in this study.
